# Machine learned daily life history classification using low frequency tracking data and automated modelling pipelines: application to North American waterfowl

**DOI:** 10.1186/s40462-022-00324-7

**Published:** 2022-05-16

**Authors:** Cory Overton, Michael Casazza, Joseph Bretz, Fiona McDuie, Elliott Matchett, Desmond Mackell, Austen Lorenz, Andrea Mott, Mark Herzog, Josh Ackerman

**Affiliations:** 1 Western Ecological Research Center, U.S. Geological Survey, Dixon Field Station, Dixon, CA USA; 2 Cloud Hosting Solutions, U.S. Geological Survey, Bozeman, MT USA; 3 Moss Landing Laboratories, San Jose State University Research Foundation, San Jose, CA USA

**Keywords:** Animal behavior, Daily activity, Life history state, Global positioning system, Supervised machine learning, Classification, Automated model pipeline, Biologging, Waterfowl, Anatidae, Telemetry, Daily activity routine

## Abstract

**Background:**

Identifying animal behaviors, life history states, and movement patterns is a prerequisite for many animal behavior analyses and effective management of wildlife and habitats. Most approaches classify short-term movement patterns with high frequency location or accelerometry data. However, patterns reflecting life history across longer time scales can have greater relevance to species biology or management needs, especially when available in near real-time. Given limitations in collecting and using such data to accurately classify complex behaviors in the long-term, we used hourly GPS data from 5 waterfowl species to produce daily activity classifications with machine-learned models using “automated modelling pipelines”.

**Methods:**

Automated pipelines are computer-generated code that complete many tasks including feature engineering, multi-framework model development, training, validation, and hyperparameter tuning to produce daily classifications from eight activity patterns reflecting waterfowl life history or movement states. We developed several input features for modeling grouped into three broad categories, hereafter “feature sets”: GPS locations, habitat information, and movement history. Each feature set used different data sources or data collected across different time intervals to develop the “features” (independent variables) used in models.

**Results:**

Automated modelling pipelines rapidly developed easily reproducible data preprocessing and analysis steps, identification and optimization of the best performing model and provided outputs for interpreting feature importance. Unequal expression of life history states caused unbalanced classes, so we evaluated feature set importance using a weighted F1-score to balance model recall and precision among individual classes. Although the best model using the least restrictive feature set (only 24 hourly relocations in a day) produced effective classifications (weighted F1 = 0.887), models using all feature sets performed substantially better (weighted F1 = 0.95), particularly for rarer but demographically more impactful life history states (i.e., nesting).

**Conclusions:**

Automated pipelines generated models producing highly accurate classifications of complex daily activity patterns using relatively low frequency GPS and incorporating more classes than previous GPS studies. Near real-time classification is possible which is ideal for time-sensitive needs such as identifying reproduction. Including habitat and longer sequences of spatial information produced more accurate classifications but incurred slight delays in processing.

**Supplementary Information:**

The online version contains supplementary material available at 10.1186/s40462-022-00324-7.

## Background

Understanding an animal’s movement, behavior, and resulting demographic outcomes requires understanding the life history context of the observed patterns, because individual life history states reflect specialized resource needs, resource quality, or produce distinct impacts to an individual’s fitness [1–5]. Thus, an individual’s life history state provides important context to understand resource selection, survival, reproduction, and distribution patterns throughout an individual’s lifetime [6]. Time-sensitive research actions, such as confirming nesting site and fate or animal mortality and cause, would benefit from rapid classification of daily activities linked to life history-specific behavior patterns of marked individuals. Meanwhile, the inability to accurately and rapidly identify daily activity when important life history events last for short periods, such as nests which fail during laying or early in incubation, may lead to biased ecological interpretations [3, 7, 8]. Furthermore, near real-time classification of animal life history states from marked individuals would be advantageous for crucial management endeavors such as abatement programs designed to minimize conflicts with migrating animals [9] or disease surveillance efforts [10, 11]. Since animal behavior often differs according to individual life history state needs, and behavior is expressed through patterns of movement, we can use movement to classify divergent behavior [12–14] and differentiate among activities related to specific life histories. Such movement information is obtainable by electronically tracking organisms with Global Positioning Systems (GPS).

Techniques to categorize animal behavior using GPS locations, accelerometry, and other methods (see [15, 16]) have proliferated in recent decades as animal-borne sensors have become lighter, less expensive, and capable of obtaining greater quantities and types of information [17]. Ecological applications of GPS tracking have benefited from techniques developed to analyze increasingly ubiquitous human-borne sensors present in mobile phones [18] such as accelerometry, which allows behavioral classification using supervised, unsupervised, or clustering methods [19]. Suitability among these approaches for a particular use-case depends on the pattern of data acquisition, i.e., continual collection at low frequency [20] or episodic collection at higher frequencies [21], and whether a priori knowledge of relevant behavior or movement classes exist and are available to label data elements which are required for supervised classification methods. Each of these approaches typically identify and cluster relatively homogenous patterns and then classify the short-term movements or inferred behaviors [1].

Animal movement can be expressed as a hierarchy of scale-dependent units ranging from sub-second duration homogenous movements (Fundamental Movement Elements [22]) such as the down-flap of a wing or the lifting of a foot. Sequences of Fundamental Movement Elements represent behaviors or actions that occur over longer and variable timeframes ranging from several seconds to hours, (Canonical Activity Modes, CAMs [16] or “movement phases” [1]) such as flight, or walking. The set of CAMs that occur across 24 h reflect a Daily Activity Routine (DAR), which themselves combine to reflect larger scale activities extending to life history states and ultimately the lifetime track of an individual [16]. Most approaches seeking behavioral classification or segmentation of data streams from animal-borne sensors focus on CAMs [2, 22, 23], because CAMs reflect activities that are often of ecological interest (e.g., resting or foraging [2, 22]) and are homogenous enough to produce accurate groupings. CAMs may be inferred using high frequency GPS data or very high frequency tri-axial accelerometry, though classifications based on GPS location data alone do not generally perform as well as models based on accelerometry data [24]. Data from GPS loggers are constrained by device size, battery capacity, and longevity and are not capable of providing as much data to classification problems as accelerometers. Therefore, approaches using GPS data are generally limited to binary classifications (e.g., migrating vs non-migrating [15] or nesting vs not-nesting [3]) or multi-class classification with very distinct movement characteristics such as not moving, terrestrial movement, or flight [24]. This also explains why the most data intensive classification approaches, such as machine learning, investigate relative short-term behaviors (resting, feeding, flying) using high frequency accelerometry data. These constraints can limit advanced analytical methods to species that can carry larger devices able to collect higher frequency data, or to shorter time frames that reflect a small portion of an animal’s life history [16, 17]. Although these approaches are both improving and useful in many contexts, often research applications or management needs require the identification of animal activities that are complex and occur over longer durations such as nesting or molting. These larger scale behaviors are typically inhomogeneous movement patterns consisting of multiple CAMs [22] and would require unmanageable data sequences to investigate using very high frequency accelerometer data and may be better ascribed to DARs.

The aim of this study was to develop a more comprehensive and effective method for classifying longer-term, behaviorally-heterogenous, life history states (i.e., DARs) using low-resolution GPS location data. Our approach uses supervised classification with machine-learned models created using computer-generated code, hereafter “automated modelling pipeline”, that produces multiple candidate models from different modelling frameworks. The automated modelling pipeline includes data engineering steps to preprocess input data, model training (i.e., inferential model optimization), hyperparameter tuning (i.e., learning process optimization), model comparison, and optional on-line endpoint hosting to enable future, near real-time classification of novel data.

We tested the utility of automated model pipelines by using this approach to develop machine learned models that classify daily activity patterns reflecting the complete annual cycle of common North American dabbling ducks (Anatidae) and using low frequency (hourly) GPS location data obtained from 5 species: Northern Pintail (*Anas acuta*), American Wigeon (*Anas americana*), Cinnamon Teal (*Anas cyanoptera*), Mallard (*Anas platyrhynchos*), Gadwall (*Anas strepera*). The classification identifies 8 daily activities associated with life history states, including nesting, molting, and migration, as well as general movement patterns unaffiliated with these states, such as large-scale relocation within the landscape, and ambiguous movements such as semi-stationary (molt-like) activity. We assessed multiple machine learning classification frameworks and evaluated performance of models trained using combinations of 3 feature sets: target date GPS information only, arrangement of target date locations with locations during previous time periods, and remotely sensed habitat characteristics at GPS locations.

## Methods

Commercial software and modelling packages for open-source programming languages have improved access to machine learning methods to non-experts, however much of the knowledge required for efficient machine learned modelling is not possessed by many ecologists. In general, traditional modelling workflows contain several steps (Additional file 1: Table S1) that require ecological domain knowledge for data collection, preprocessing and feature development and require data science domain knowledge for effective model formulation, validation, optimization, and evaluation. The availability of automated modelling pipelines to guide machine learning workflows can substantially reduce the number and breadth of non-ecological decisions that need to be made and increase the potential application of powerful methods for prediction and classification in ecology.

A machine learning workflow for classification problems begins with data acquisition and quality control, target class identification, followed by labelling or “annotation” of known life history states present in collected data. After these steps, it is necessary to identify characteristics, or features, of the data that may be useful to distinguish between alternate classes. This process of feature development, also called feature engineering, is analogous to independent variable creation and is a crucial step in determining the ultimate performance of models particularly when limited input data is available to distinguish between complex and similar classes. However, features used for a specific use-case may not be effective at discriminating among novel classes or for other taxa or data types. The features we developed to classify daily activities of waterfowl are provided in the Additional file 1 (Table S2–S4) but we caution that species behavior and habitat affinities may limit the generalizability of these features to other models developed for other taxa.

There are important considerations for feature engineering which may improve model performance. Principle among these is the concept of data leakage. Data leakage occurs when “information” is shared between the training and validation data subsets and results in inflated assessment of model performance and poor generalizability to novel datasets. There are two steps in the machine learning workflow where data leakage can occur: during annotation of training data and during feature engineering steps. If the same features or characteristics are used during annotation to verify class assignment and during model fitting and validation, then models will tend to have higher accuracy but low generalizability could result (i.e. model overfitting) because there is a lack of independence between variables used to model classes and the process of defining representative classes of data. Additionally, data leakage during the feature engineering step may result if training data is spatially or temporally correlated and not representative of broader ecological conditions. For example, it is very difficult to obtain a random sample of nesting activity from all individuals in a population with a global distribution. Therefore, training data often relies on data obtained from focused studies undertaken in a limited portion of the species range. Using geographic coordinate information from those spatially biased nesting data would impart information to machine learned classification models that only represents a small subset of the potential nesting range for other members of the species. In extreme cases, the resulting models would only be able to identify nesting where the original training data was obtained and any nesting activity outside that study area would be misclassified. Therefore, prior to calculating spatial features, geographic coordinates of GPS locations can be mean-centered, which spatially “anonymizes” the data to reflect relative position. This will prevent spatial bias in trained model(s) and foster generalization to regions unrepresented in training data.

Following feature development and data formatting, we performed our machine learning modelling steps within Amazon Web Services’ (AWS; Seattle, WA) SageMaker Studio© (https://aws.amazon.com/sagemaker/studio/), an integrated development environment, that uses SageMaker Autopilot© (https://aws.amazon.com/sagemaker/autopilot/) [25] and a graphical user interface to rapidly develop and execute python code (see Additional file 3), thus automating many machine learning processing steps (Additional file 1: Table S1). The automated pipeline developed 10 candidate models using multiple machine learning frameworks. Frameworks assessed for our evaluation included 4 models using extreme boosted gradient descent (XGBoost; URL: https://github.com/dmlc/xgboost) [26], 5 models using AWS’s “LinearLearner” an MXNET-based stochastic gradient descent (https://docs.aws.amazon.com/sagemaker/latest/dg/linear-learner.html), and a single model using a multi-layered perceptron [27].

Performance of machine learning models can be improved through optimizing two different sets of parameters. Firstly, the inferential model uses parameters that describe how features (independent variables) relate to the daily activity class (dependent variable). It does this by “learning” how many successive evaluations of different parameter estimates improve the classification of training data without worsening classification of validation data. Secondly, machine learning algorithms use “hyperparameters” that dictate precisely how the model “learns” or improves on successive iterations. Both optimization routines evaluate model performance using an evaluation metric, often accuracy, precision, or recall. However, optimizing models with classes that are not equally represented among the training data can result in poor generalizability or decreased performance of rarer, often more important, classes [28]. Since life history states do not all occur for equal periods and/or may be limited to individual sexes, it is likely that any available labelled training data would have unbalanced class representation. Furthermore, the most demographically important life history states, e.g., nesting and care of young, are among the least readily observed activities but may often require the most accurate classification. Accuracy is affected strongly by class imbalance and may not be the most useful measure of model performance. Where training data is not balanced among possible classes, F1-score evaluation metrics—calculated as the harmonic mean of precision, the proportion of predicted cases that are classified correctly, and the model recall, the proportion of actual cases that are classified correctly—may result in more useful interpretation of model performance.

An additional important impact that hyperparameters have in machine learned models is to improve model generalizability. Machine learned models fit functions with many parameters to data which tends to result in overfitting. Hyperparameters, such as “L1” and “L2” regularization parameters, reduce the performance of the model on the training dataset in exchange for improvement on validation datasets. This reduces overfitting and increases generalizability. Therefore, hyperparameters contain no biological information relevant to the classification problem, but govern model complexity, the rate of improvement, and other mechanistic aspects of the modelling. Hyperparameter ranges evaluated for our case study are provided in the Additional file 1 (Table S5).

### Waterfowl daily activity classification case study

The goal of our case study was to build flexible daily behavioral classification models suitable for multiple dabbling duck species (*Anas* sp.). Such models, which can be applied to multiple taxa, are useful because they can reduce the need to produce many individual species-specific models, however, the efficiency of multispecies models may result in reduced accuracy if species-specific heterogeneity in behaviors exist. North American waterfowl are an ideal taxon to produce a multispecies model because most dabbling duck species exhibit similar activities at similar times of year which allows efficient labelling of movements and behaviors into recognizable life history states. Most species of dabbling duck have relatively fast life history traits, such as large clutch sizes and precocial young. And most species demonstrate solitary nesting and prolonged care of precocial young (brooding) by females. Both sexes experience periods of flightlessness during a complete molt of primary feathers in late summer. Many species exhibit seasonal migratory behavior [29] including post-breeding migrations to molting areas, but individual populations may also be nonmigratory or express mixed migration strategies [30, 31].

**Fig. 1:**
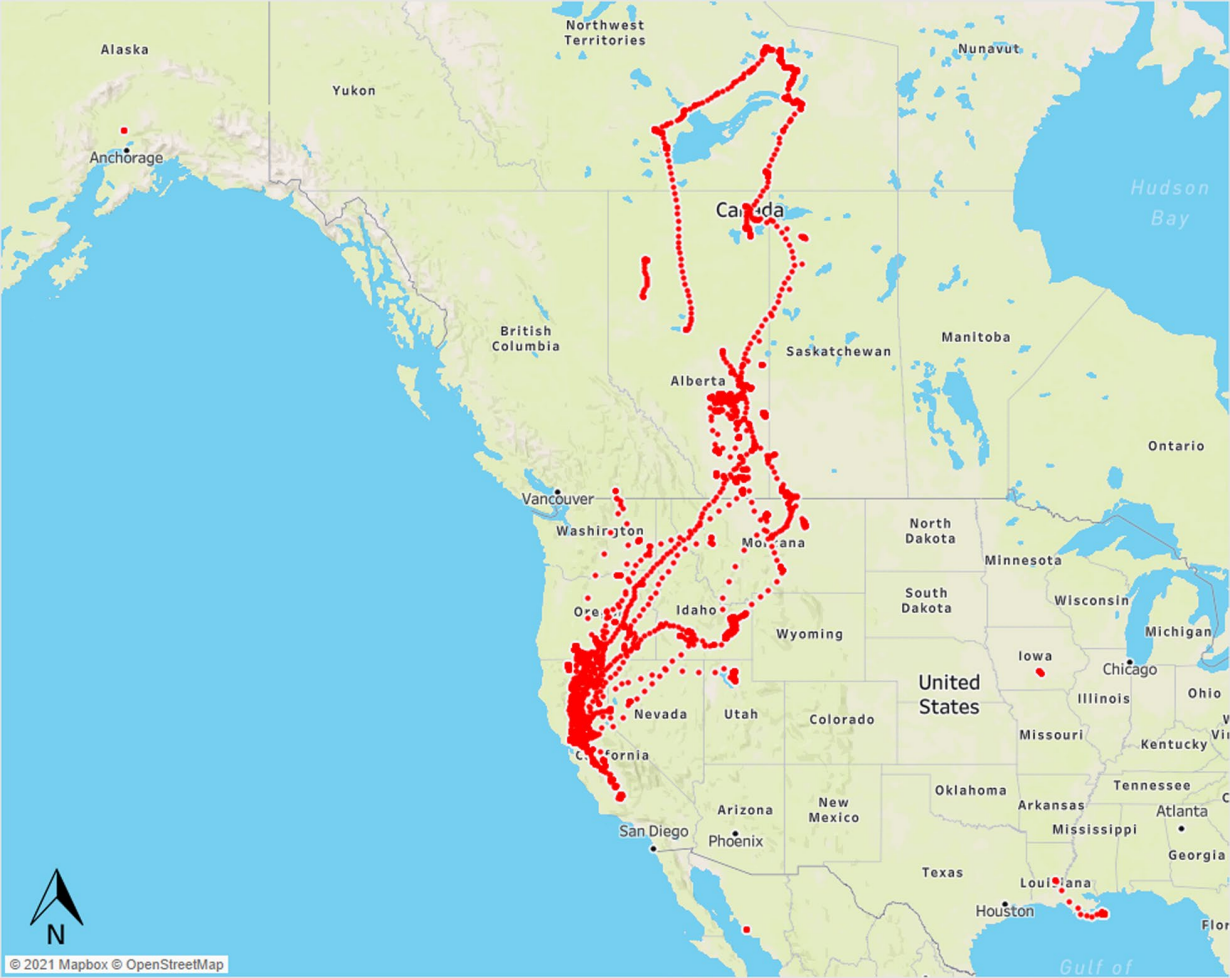
Extent of 224,016 GPS locations obtained from 131 individual ducks of 5 species and representing 9334 bird-days. Daily sets of hourly location data used to train and validate machine learned classification models for dabbling duck life history states and movement patterns

Data used in our case study was built on previous studies describing DARs for ducks occupying California’s Central Valley (see [12] for details on capture methods and study area). Location data were obtained from 131 marked dabbling ducks representing 5 species: Mallard (*Anas platyrynchos*), Gadwall (*A. strepera*), Northern Pintail (*A. acuta*), Cinnamon Teal (*A. cyanoptera*), American Wigeon (*A. americana*; Table 1). We used locations obtained from 5000 bird-days at hourly, half-hourly, or 15-min intervals between January 2015 and August 2020 (Fig. 1). Data were assessed for positional errors resulting in the exclusion of one bird-day due to incomplete transmission of coordinate data resulting in 4999 sets of 24-h GPS location data being used to model daily activity routines of North American waterfowl (Table 2). We used data augmentation procedures that subset higher frequency data into constituent hourly sets [32], such that half-hourly data provided two independent DARs and 15-min interval GPS location provided four independent DARs for modelling. Therefore, our final set of data included 9334 bird-days of hourly GPS locations from which to develop features for model training, validation, and testing (Table 2; see Overton et al. [33] for data availability). Details of field procedures, marking, and data processing are provided in McDuie et al. [12, 14] and the Additional file 3.

**Table 1:** Distribution of 8 life history states or movement patterns used to train and validate machine learned classification models

	Brooding	Dead	Local	Migration	Molt-like	Molting	Nesting	Regional relocation
Northern Pintail	0	54	1238	64	422	56	0	103
American Wigeon	0	0	0	4	12	0	0	0
Cinnamon Teal	8	522	188	3	166	6	102	9
Mallard	41	1	1914	4	1466	190	107	39
Gadwall	56	0	971	24	896	184	80	34
Undeployed	0	370	0	0	0	0	0	0

Annotation was performed on daily sets of 24 hourly GPS locations obtained between January 2015 and August 2020 from 131 free-living waterfowl representing 5 species in North America, includes GPS locations from two undeployed transmitters representing bird mortality

**Table 2:** Input data elements consisted of 24 GPS locations collected hourly within a single day

Data frequency	Number of bird days	Number of augmented (hourly) data elements
Hourly	2260	2260
Half-hourly	1941	3882
Quarter-hourly	798	3192
Total	4999	9334

Higher frequency locations were subset to consistent hourly sets to augment available training data. Data collected between January 2015 and August 2020 from 131 free-living waterfowl representing 5 species in North America, includes GPS locations from two undeployed transmitters representing bird mortality

**Fig. 2:**
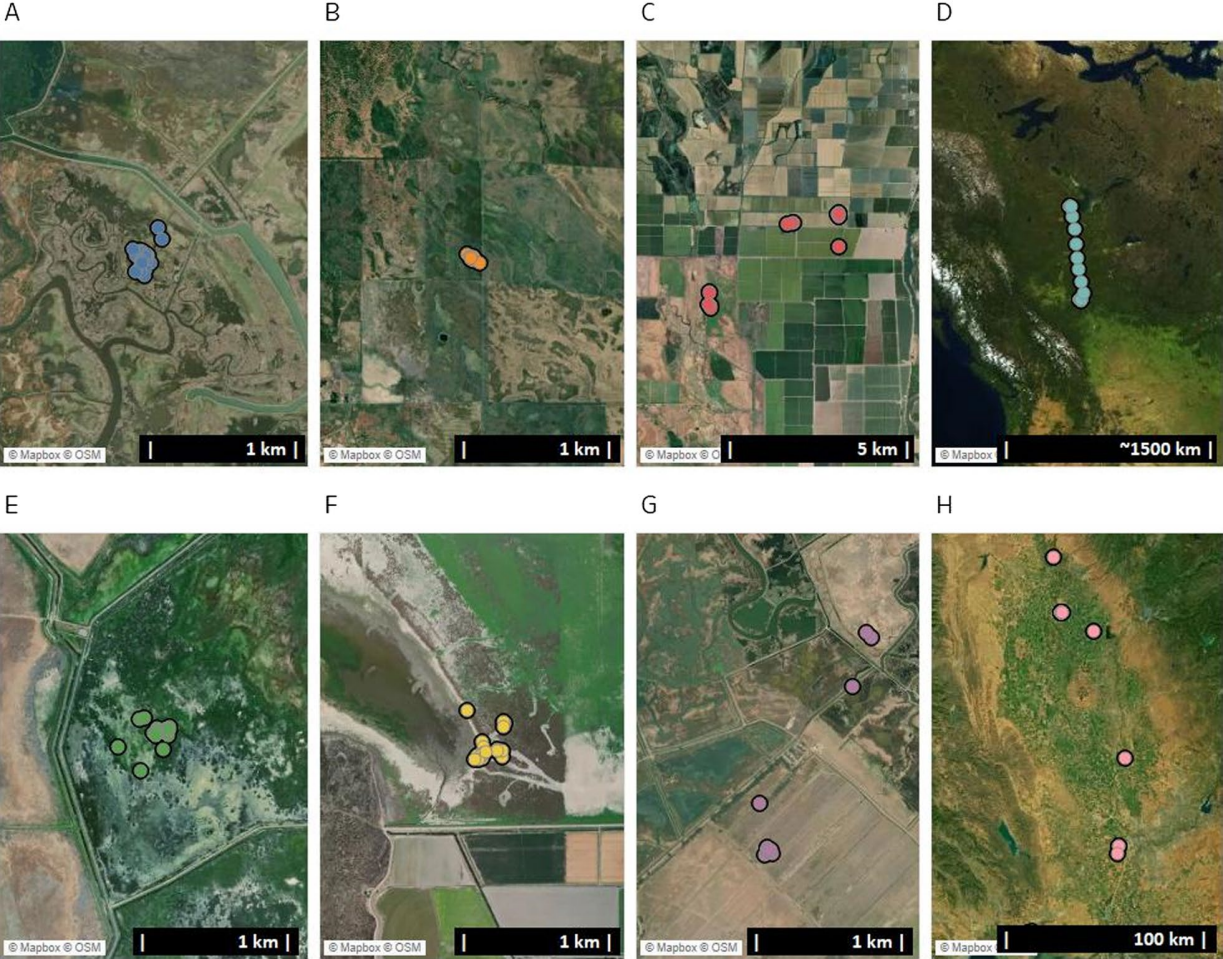
Each daily set of hourly GPS locations were classified into 8 life history categories representing the daily activities of waterfowl including: **A** brooding; **B** dead; **C** local movements; **D** migration; **E** molt-like movements; **F** molting; **G** nesting; **H** regional relocation movements

We annotated daily activity into 8 mutually exclusive life history states (Fig. 2, Table 1) using independent data [34, 35] or algorithmic identification of activity [24, 30] to develop preliminary classifications which were verified visually by waterfowl biologists using supplemental information on individual fate and longer sequencies of location data. Four classes reflected phenologically-mediated life history states: nesting, brooding, molting, dead. The remaining four classes reflected more general movement patterns occurring outside these biologically-constrained life history states: molt-like, local movements, regional relocation, and migration. Descriptions of each life history state and detail on annotation methods are available in the Additional file 2.

Following annotation of known data classes, we engineered features for use in the machine learned models by generating meaningful characteristics or summaries from complex raw data (e.g., median hourly movement distance or total daily displacement). We developed 68 total features using three different types of information derived from each day’s GPS locations (see Additional file 1 for a complete list and details). The primary feature set consisted of 40 features derived only from the spatial position and timing of hourly GPS locations, hereafter termed the “movement and timing” feature set. These features described characteristics of movement or space use (e.g., step length/rate of movement, displacement, space use) often with specific reference to photoperiod (e.g., daytime or nighttime dispersion of locations). The second feature set used remotely sensed satellite information at location coordinates to reflect flooding condition of habitats used when the bird occupied an area, we refer to these as the “habitat” feature set. Eight features were developed that used the average Modified Normalized Difference Water Index (MNDWI) [36] derived from Landsat-8 and Sentinel-2 imagery collected during the same month that locations were obtained and composited using Google Earth Engine [37]. The last feature set consisted of 20 metrics derived from the spatial arrangement of target date locations relative to GPS location data obtained either 1 day, 2–3 days, 5–7 days, 8–10 days, or 12–15 days before the target date. We refer to these as the “history” feature set. After calculation of all features, the 9334 available data records were randomly assigned to 3 data sets for model training (64% of available records), validation (16% of available records), and testing (20% of available records). All feature sets were developed in R version 3.6.0 [38] interfaced with Google Earth Engine [37] to calculate monthly MNDWI values from satellite images. Packages used to develop features are provided in Additional file 2.

Each feature set entailed a different set of limitations for modelling. Movement and timing features were the least restrictive, requiring only a complete set of 24 hourly GPS locations, which could enable classification within minutes of data transmission from the bird-borne transmitter. The habitat feature set resulted in the longest delay in producing classifications because remotely sensed imagery had to be processed and transmitted to Google Earth Engine before features could be developed. This results in approximately a 72-h delay between GPS location acquisition and calculation of concurrently collected remote imagery. The history feature set entails the greatest limitation regarding the suitability of input data, because it required continuously collected data for 15 days prior to the date being classified. This continuous data requirement means transmitters that have voltage dependent scheduling or periods our missing data would need to be excluded from classification due to the inability to calculate all features required by the model.

Data preprocessing steps such as regularization, and model training, model validation, and model evaluation were performed automatically by code generated by the automated pipeline. The automated pipeline also generated code that performed a Bayesian search to “tune” hyperparameters within a range of potential values 200 times among all candidate models. Code produced by the automated pipeline and description of preprocessing steps and hyperparameter tuning ranges are provided in the Additional file 4. We modified this code to conduct the Bayesian hyperparameter tuning 200 times for each model rather than among all models to ensure equal effort was expended on each candidate model algorithm. Model training and hyperparameter tuning was optimized using 64% of available data and validated with 16% of available data. Due to limitations in native programming capabilities, the automated pipeline trained models and tuned hyperparameters using the macro-F1 score (or class averaged harmonic mean of model recall and precision) as the evaluation metric. However, macro-F1 scores do not account for class imbalance in training data. Therefore, after model training and hyperparameter tuning, we tested the final optimized model for each candidate pipeline with the remaining 20% of data withheld from all prior analyses and subject to the same preprocessing steps. From these results we produced confusion matrixes and calculated the class weighted F1-score (hereafter, “weighted-F1 score”) [39] to compare the performance of the best trained and tuned model from each model pipeline. Alternate use cases may require different evaluation metrics, so we present a suite of commonly used metrics (e.g., precision and recall) in our results. We refit the best performing candidate model pipeline that was developed using all 3 feature sets using only the movement and timing feature set, the movement and timing feature set combined with habitat feature set, and the movement and timing feature set combined with the history feature set. This resulted in 4 final model pipelines each reflecting different limitations regarding data constancy or delays in producing classifications.

## Results

Automated modelling pipelines rapidly developed data preprocessing and analysis code that evaluated 10 candidate models using 3 machine learning frameworks, identified and optimized the best performing model and provided outputs for interpreting feature importance. Automated pipelines produced code that could be modified to achieve project-specific needs such as hosting the model to endpoints to provide batch or real-time classification of novel data or producing graphics or summaries of model performance such as confusion matrices of final classifications. The pipeline applied to waterfowl daily activity classification includes feature processing that can impute missing data, but missing data was not present in our training dataset so this step resulted in no changes for our case study. Additional preprocessing steps were identified for candidate models, including scaling, and centering numeric features, Principal Component Analysis (PCA) dimension reduction, and threshold indicator variable encoding (also called “one-hot” encoding) categorical variables or “sparse” continuous variables that had few discrete values. The set of preprocessing steps, including selection of threshold values for indicator variables, were uniquely applied to separate models using the same machine learning framework (i.e., the 4 XGBoost and 5 LinearLearner models; Table 3). All transformations were applied after the splitting of data between training, testing, and validation datasets which prevents the phenomenon of “data leakage” from occurring. The modified hyperparameter tuning approach we used to assess each model equally identified the same best performing candidate model as automated code which performed a simultaneous Bayesian search among all models. Our best model and the model identified by the original automated code had nearly identically tuned hyperparameter values and produced identical classifications on both validation and testing data.

**Table 3:** Candidate model pipeline framework and data transformation steps produced by SageMaker Autopilot©

Model #	Framework	Data transformation steps
1	XGBoost	Create Threshold One Hot Encoding (threshold = 30) for categorical/sparse features
2	LinearLearner	Converts features with extreme values to a uniform distribution Feature dimension reduction using PCA
3	LinearLearner	Scaling and centering features while accounting for data sparsity only
4	XGBoost	Create threshold one hot encoding (threshold = 5) for categorical/sparse features
5	LinearLearner	Create threshold one hot encoding (threshold = 6) for sparse features Feature dimension reduction using PCA
6	LinearLearner	Create threshold one hot encoding (threshold = 7) for categorical/sparse features
7	LinearLearner	Create threshold one hot encoding (threshold = 7) for categorical/sparse features Feature dimension reduction using PCA
8	XGBoost	Create threshold one hot encoding (threshold = 7) for categorical/sparse features
9	XGBoost	Create threshold one hot encoding (threshold = 9) for categorical/sparse features
10	MLP	Scaling and centering features while accounting for data sparsity only

Data processing steps utilize functions from the AWS ScikitLearn extention (https://github.com/aws/sagemaker-scikit-learn-extension, copyright AWS 2019). Models represent 3 frameworks: Extreme Gradient Descent (XGBoost); Stochastic Gradient Descent (aka LinearLearner); and Multi-Layered Perceptron. Data transformation for each candidate pipeline automatically included imputation of missing values which were not present in training data. Each candidate model included a processing step to scale and center features while accounting for data sparsity

Classification accuracies of the daily activity of waterfowl among our models were moderate to high (micro-accuracy 0.818-0.952) but may be misleading due to imbalanced life history state classes. Therefore, we relied on evaluation using a weighted F1-score that balances model recall and precision among individual classes which were similar to the overall accuracy scores (0.811–0.950; Table 4). The best performing model was an XGBoost classification with on-hot encoding (synonymous with a “binary indicator variable”) generated for sparse valued features using a threshold value of 30 (Model 1; Table 4). When evaluated against withheld testing data, the weighted-F1 score was 95.0%. Overall, the accuracy was 95.2% and the macro-F1 score was 89.9%, slightly lower than the value calculated against the validation data during model training (92.4%). Class-specific F1-scores exceeded 85% for 7 of the 8 daily activity classes with only “Brooding” falling below that level (Table 5). Confusion matrices for all other candidate model pipelines are provided in the Additional file 1. When fewer feature sets were used to train classification models, model performance declined but declines were modest for some daily activity classes (e.g., Dead and Local movements). Model performance patterns indicated that classification among all classes was improved with the inclusion of all feature sets, additional feature sets, and individual features with movement only information, except that classification of brooding was not improved by the addition of the history feature set relative to the absence of those features (Table 6). In general, class predictions were better when we included only the history feature set (weighted F1 = 0.928) compared to including only the habitat feature set (weighted F1 = 0.924), except for classification of the Migration class. Brooding was poorly predicted for all model pipelines and feature combinations and was most frequently classified as the heuristically similar molt-like movement pattern (see Additional file 1).

**Table 4:** Performance metrics for 10 candidate model pipelines (Model Numbers from Table 3) classifying daily activity of waterfowl into 8 classes using GPS-derived feature datasets reflecting movement and timing, habitat, and history of movement

Evaluation metric	Model number (%)									
	1	2	3	4	5	6	7	8	9	10
Accuracy	95.2	86.4	86.7	94.8	85.8	81.8	85.5	94.8	94.9	92.4
Macro-precision	96.3	76.3	80.7	96.3	76.7	70.3	75.4	96.3	96.3	86.5
Macro-recall	87.1	71.6	72.5	86.7	69.9	63.4	70.0	86.7	87.2	82.9
Macro-F1	89.9	73.3	74.6	89.7	72.1	65.8	71.8	89.7	89.9	84.1
Weighted-precision	95.3	85.7	86.4	94.8	85.3	80.9	84.7	94.8	94.9	92.3
Weighted-recall	95.2	86.4	86.7	94.8	85.8	81.8	85.5	94.8	94.9	92.4
Weighted-F1	**95.0**	86.0	86.3	94.6	85.4	81.1	84.9	94.6	94.7	92.2

Due to class imbalance, we determined the best performing model using the weighted-F1 score, in bold

**Table 5:** Confusion matrix and class specific performance metrics of the best performing, optimized, model pipeline using all three feature sets (movement and timing, habitat, and history) to classify daily activity of waterfowl into 8 classes

Actual class	Predicted class								F1-score	Precision	Recall
	Brood	Dead	Local	Migration	Molt-like	Molting	Nesting	Regional relocation			
Brooding	8	0	2	0	11	0	0	0	0.552	1.000	0.381
Dead	0	189	0	0	0	0	0	0	1.000	1.000	1.000
Local	0	0	839	0	20	0	0	3	0.969	0.964	0.973
Migration	0	0	0	19	0	0	0	1	0.974	1.000	0.950
Molt-like	0	0	27	0	561	3	2	0	0.932	0.918	0.946
Molting	0	0	0	0	14	73	0	0	0.896	0.961	0.839
Nesting	0	0	2	0	5	0	51	0	0.919	0.962	0.879
Regional relocation	0	0	0	0	0	0	0	37	0.949	0.902	1.000

**Table 6:** Class specific F1-scores and overall weighted F1-score across all classes (in bold) from best performing model using different combinations of available feature sets

	All feature sets	Movement and timing and habitat features	Movement and timing and history features	Movement and timing features only
Brooding	0.552	0.240	0.000	0.000
Dead	1.000	0.992	0.997	0.984
Local	0.969	0.954	0.965	0.946
Migration	0.974	0.947	0.974	0.923
Molt-like	0.932	0.899	0.909	0.856
Molting	0.896	0.824	0.764	0.577
Nesting	0.919	0.899	0.897	0.792
Regional relocation	0.949	0.923	0.935	0.895
**Weighted-F1**	**0.950**	**0.924**	**0.928**	**0.887**

## Discussion

We found that automated model pipeline generation evaluating multiple machine learning frameworks and data preprocessing transformations can accurately and precisely classify complex and heterogenous behaviors at biologically relevant, short-term (daily), time scales while using features engineered from only relatively low frequency (hourly) GPS data even though available training data was unbalanced. Our empirical study classified 8 life history states or movement patterns among a suite of waterfowl species in North America and indicates high predictive accuracy and precision (weighted F1-score > 90%) for most classes. Our classification accuracy and precision were substantially greater compared with previous studies of life history classification and improved with the inclusion of additional features reflecting habitat and historical position information. Shamoun-Baranes et al. [24] classified eight behavior classes that are reflective of CAM behaviors (e.g., standing, foraging, flying, walking) with both accelerometry and GPS data (error rate of 28%) but were only able to discern three classes (not moving, flying, terrestrial movement) with GPS data only, for which they obtained an error rate of 33%. By contrast we assessed classes that were expressed over longer time frames (DARs), consisted of multiple heterogenous behaviors, and used comparatively low-resolution GPS data only, yet we reached average class accuracies with error rates below 8% using only GPS data (when including location history features) and below 5% when also including habitat information. Three factors resulted in the greater performance of our models to the previous efforts. First, we used various types of data to engineer features relevant to waterfowl life history states including spatial arrangement of locations, habitat, and/or spatial arrangement of locations for a target date to be classified to locations collected on prior days. Whereas the incorporation of different types of data did improve model performance, even models using only moderate resolution GPS locations obtained higher accuracies than prior efforts. This appears largely related to developing features that are particularly useful and distinguishing among similar movement patterns (Fig. 2) and the preprocessing steps and assessments of multiple model frameworks initiated by the automated modelling pipeline.

Although feature engineering is specific to each classification problem and dataset, often the more features which can be applied to a classification problem, the better a model will perform. Shamoun-Baranes et al. [24] has the same number of classes as we did and used 1 GPS-derived feature and 13 accelerometry derived features. We developed 40 features just from the hourly locations collected on the target date and 28 additional features representing habitat condition or prior GPS locations (see Additional file 1). Leveraging the information present in additional features, in combination with the preprocessing steps and multi-model assessments completed by the automated modelling pipeline, resulted in substantially higher model performance.

In addition to obtaining greater classification accuracies using GPS data collected at moderate frequencies, another advantage to our approach is the use of inexpensive cloud-based commercial services that allow results to be deployed locally or in the cloud for distributed and near real-time classification, making immediate information broadly available. Novel data collected at other locations or times that undergo the same feature development steps may be fed into our pre-trained models to produce real-time classifications. Given the capacity of modern GPS loggers to transmit data via cellular networks [17], final classifications can occur within hours of collection.

Many existing applications of machine learning using wildlife movement data focus on identifying homogenous short-term movements or stationary processes from animal relocation data (e.g., fundamental movement elements [22, 40, 41]). Quite often, high frequency accelerometry is used with, or in place of, GPS location data [42–45]. However, upscaling fine-scale behaviors or movement patterns to other biologically relevant longer-term and more heterogenous patterns remains elusive [23]. Most current efforts to do so limit the classification problem to a binary framework [3, 15] which requires application of multiple different models to classify a complete annual life history cycle. However, hierarchical modelling may compound inaccuracies in prediction due to error propagation where misclassifications in earlier models cannot be rectified in subsequent models.

Many aspects of animal life history and associated behaviors reflect either long-term processes or occur sequentially. This suggests that machine learned classifications may ultimately be improved through either post-hoc assessment or the inclusion of sequential life history state progression in modelling efforts. Among our case study for example, waterfowl brooding activity was the least commonly occurring life history state and consequently the least represented among labelled training data. Brooding is also very similar to other classes of activity (Fig. 2) making the classes difficult to distinguish from each other. But brooding also must chronologically follow nesting activity. Given the nearly equivalent accuracies produced by the multi-layer perceptron model to the best performing XGBoost model in our empirical example (weighted F1-score = 0.922 vs 0.950, respectively), we expect that more “temporally aware” sequence-dependent prediction frameworks, such as Long Short-Term Memory methods [46], may improve predictability when class assignments follow in a logical progression [50]. Similar methods have been used to reveal animal migration strategies [47] but were not yet implementable within Amazon SageMaker AutoPilot© at the time of our investigation. “Super-learning” or ensemble methods that combine and optimize results from multiple models may likewise improve model performance as they have for accelerometry-based classifications [43].

As our empirical modeling results demonstrate, selection of feature sets used in modelling affect accuracy, recall, and precision of models and may impact individual classes differently. Depending on end-use case needs, this can present a tradeoff between model accuracy and data consistency requirements or delays in prediction as auxiliary data is prepared (e.g., satellite imagery processing). Thus, operational objectives may require the use of “sub-optimal” models that enable near real-time classifications. Examples may include tracking the spread of active disease outbreaks involving wild species as vectors [48], mortality, nest monitoring [49, 50], and proximity- or behavior-based wildlife warning, abatement, or management actions [51, 52]. When delay in model predictions is not acceptable, a lower accuracy classification may still enable more efficient deployment of personnel or resources to meet specific end-user needs. Understanding these tradeoffs from classification strategy (near-real time, but lower performing models versus delayed, but higher accuracy classifications) will inform interpretation of results and enable appropriate responses based on observed class-specific accuracies. For instance, it may not be efficient to devote resources to confirm molting activity identified from only daily GPS location data because error rates exceeded 40% for that class, but where data is consistent enabling spatial comparison to previous locations, then error rates are reduced to nearly 25% and should result in more efficient allocation of personnel. As such, evaluation metrics are useful for identifying classes lacking reliable prediction and may be used to assess whether additional feature sets improve overall performance. For our research, we developed two additional feature sets extending beyond the characteristics of the 24 hourly GPS locations themselves; habitat information and spatial arrangement of current position with prior locations. Including these feature sets in models, substantially improved classification performance for molting (0.32 greater F1-score) and nesting (0.12 greater F1-score) life history states (Table 6). Classification of brooding also improved greatly when all three feature sets were included, although overall accuracy remained low enough to warrant investigation into additional possible features that may improve brooding classification.

## Conclusions

In this manuscript, we describe the use of automated model pipelines to develop and evaluate multiple machine-learned models to classify daily activities related to wildlife life history states. The use of automated modelling pipelines yielded more accurate assignment of waterfowl life history and movement patterns while also involving less effort to develop code. The utility of automated modelling pipelines makes highly accurate classification possible for ecologists that may not have formal machine learning training. Broader implementation by other researchers requires feature development relevant to the taxa or life history states of interest but also that prevents classification bias, or data leakage, resulting from spatially aggregated training data. Choice of evaluation metrics for model training and tuning should consider whether the training data has class imbalance resulting from shorter, or sex-specific life history states.

Our application of automated pipelines for machine learned classification of waterfowl activity demonstrate how this approach can produce accurate daily predictions of waterfowl activity using 3 input feature sets: hourly GPS location data only, remotely sensed habitat characteristics, and arrangement of target date locations to locations from prior periods. Model performance for most classes was high suggesting these methods may be used to independently identify cryptic life history states that can reduce methodological bias in ecology studies and increase management response and wildlife surveillance and abatement options.

## Supplementary Information

**Additional file 1**. Supplementary Methods: Waterfowl data and modeling workflow (data collection, and feature engineering, hyperparameter tuning ranges) and Results (confusion matrix of alternate models from automated data pipeline).

**Additional file 2**. Feature Engineering Script for Daily Activity Classification of Waterfowl Life History States.

**Additional file 3**. Data preprocessing, testing data splitting and preparation for AWS SageMaker Studio.

**Additional file 4**. Automated Modeling Pipeline Code.

## Data Availability

Data used to develop models has been published in the U.S. Geological Survey ScienceBase Digital Repository (https://www.sciencebase.gov/catalog/) at https://doi.org/10.5066/P9XBZKZ8.

## Abbreviations

AWS: Amazon Web Services
CAM: Canonical Activity Mode
DAR: Daily Activity Routine
GPS: Global Positioning System
MNDWI: Modified Normalized Difference Water Index
PCA: Principal Component Analysis
