# Supplementary material for: Machine learned daily life history classification using low frequency tracking data and automated modelling pipelines: application to North American waterfowl
Source: Mov Ecol. 2022 May 16;10:23. doi: 10.1186/s40462-022-00324-7 (PMC9109391; doi:10.1186/s40462-022-00324-7)
Supplement: Supplementary file 1 — Additional file 1. Supplementary Methods: Waterfowl data and modeling workflow (data collection, and feature engineering, hyperparameter tuning ranges) and Results (confusion matrix of alternate models from automated data pipeline). [file 40462_2022_324_MOESM1_ESM.docx]

**Supplementary Information for:**

**Machine learned daily life history classification using low frequency tracking data and automated modelling pipelines: Application to North American waterfowl**

Cory Overton^1*^, Michael Casazza^1^, Joseph Bretz^2^, Fiona McDuie^13^, Elliott Matchett^1^, Desmond Mackell^1^, Austen Lorenz^1^, Andrea Mott^1^, Mark Herzog^1^, Josh Ackerman^1^

Additional File 1:

***Supplementary Methods: Waterfowl data and modeling workflow (data collection, and feature engineering, hyperparameter tuning ranges) and Results (confusion matrix of alternate models from automated data pipeline)***

***General machine learning processing workflow***

Machine learning workflows are complex with multiple decision points each of which will affect model performance, efficiency, or usability. We outline 13 common steps practitioners of machine learning commonly employ. Automated modelling pipelines produce code and make relevant decisions on half of those decision points (Table S1). The final workflow we used to classify waterfowl daily activities included four steps related to data preparation and dependent variable/class identification: data acquisition, quality control, labelling or annotation of known life history states among collected data, and augmenting available labeled data where greater than hourly GPS (i.e., 15- or 30-minute interval) data existed. These steps were followed by feature development, which is analogous to independent variable creation.

***Data collection***

We marked 131 marked dabbling ducks representing 5 species: Mallard (*Anas platyrynchos*), Gadwall (*A. strepera*), Northern Pintail (*A. acuta*), Cinnamon Teal (*A. cyanoptera*), American Wigeon (*A. americana*). Individuals were caught using rocket nets, swim in traps, or by long-handled nets via airboats and fit with a backpack-mounted solar-powered 10–20-gram Ornitela© or Ecotone© GPS-GSM transmitter using a neoprene-nylon ribbon harness following methods described by McDuie et al. [1, 2]. Input data for classification models consisted of continuously collected GPS locations collected at hourly intervals and segmented into 5,000 calendar date periods among all individuals (i.e., bird-days). These original 5,000 bird days are a subset of available data where at least hourly locations were obtained and for which daily activity routines could be discerned. We also removed from consideration any data where the assessed daily activity routine changed during the day (e.g., dates when nests failed) and therefore represented mixed class behaviors. Available days with data are more prevalent in spring, summer, and early fall because some life history states are restricted to those periods (e.g., nesting brooding and molting) and because shortened daylight periods, lower sun angle, and increased prevalence of inclement weather reduced solar recharge and consistency of suitable data during the winter.

***Data annotation***

Waterfowl life history states were labelled into 8 life history classes: nesting, brooding, molting, dead, migration and molt-like, local, or regional movement patterns. Molt-like movements were indicated when daily GPS locations expressed no flight activity and movements were localized within one or adjacent hydrologic basins (ponds). Local movements were often recursive and reflected typical daily roosting and feeding behavior of individuals. Regional relocation was indicated by long-distance, non-recursive flights but those that stayed within a relatively contiguous landscape. Migration occurred when very long distance non-recursive flights were taken across discontinuous landscapes. Extended stopover site use occurring within the context of longer migration periods was not classified as migration as each day is classified independently without reference to the class prediction of prior or subsequent days. Instances of extended stopover site use were typically identified as regional relocation or local type movements. Nesting was identified through independent field monitoring of nests themselves [3] and not through interpretation of bird tracks. Waterfowl broods are precocial and not cared for at the nest site, therefore the start of brooding was identified when hens and chicks left successful nests [4]. Termination of brooding, timing of molting, migratory activity and mortality were each initially determined algorithmically and subsequently confirmed by waterfowl biologists with visual support of GPS relocation paths [5, 6]. Similarly, visually supported annotation by domain experts was used to annotate dates where birds exhibited local, molt-like and regional relocation movement patterns [5]. GPS location data collected prior to deployment from two stationary transmitters were used to represent bird mortality and augment the Dead class. The processes used when labelling training data may lead to data leakage if the same features or characteristics used to identify the training data classes are also used to create classification models. We used independently collected data, previously published algorithmic methods and visual review of location data by waterfowl experts which included a longer context of individual bird’s movement, behavior and fate with which to identify classes for training data. As a result, none of the features that we ultimately used in classification models were used to annotate training data and there was no mechanism for data leakage to occur during annotation.

***Feature engineering and feature sets***

Following annotation of known data classes, we engineered features, independent variables, to use in the machine learned models, a process by which we generated meaningful characteristics or summaries from complex raw data (e.g., median hourly movement distance or total daily displacement). Features were developed while recognizing three principal considerations regarding waterfowl behavior: (1) different life history states will result in different daily activities which will express different characteristics or summary metrics of movement; (2) some life history states are associated with distinct habitat needs [7]; and (3) some life history states occur continuously for long durations. Accordingly, the features we developed to represent ecological knowledge of waterfowl may not translate to other taxa or even determination of alternate classes of behavior or movement. We developed features using the Program R version 3.6.0 [8] and 3 sets of related features were developed (Table S2-S4). The Movement and Timing feature set (Table S2) calculated features using only the spatial and temporal characteristics of 24 GPS locations comprising a data element (bird-day). These features included characteristics of diurnal and nocturnal space use patterns, because dabbling ducks often display different behaviors according to photoperiod [9, 10]. We spatially anonymized all features by mean centering latitude and longitude, where appropriate, to reflect only relative spatial position and prevent data leakage. The History feature set (Table S3) included spatial comparisons between locations collected on the target date to all locations collected during prior time periods for the same individual. The Habitat feature set (Table S4) used R to interface with Google Earth Engine [11]. Eight features were developed that used the average Modified Normalized Difference Water Index (MNDWI) [12] derived from Landsat-8 and Sentinel-2 imagery collected during the same month that locations were obtained and composited using Google Earth Engine [11]. MNDWI values greater than or equal to 0 indicated flooded habitat at the location, MNDWI values less than 0 indicated dry habitat. Individual features were developed to reflect mean and median condition and summarize MNDWI values across all 24 locations or summarize number of locations relative to differing MNDWI threshold values.

The following R packages were used to process data and develop feature sets:

`readr`, `dplyr`, `amt`, `lubridate`, `geosphere`, `maptools`, `rgdal`, `tidyr`, `readxl`, `sf`, `rstudioapi`, `stringr`[13-24]

***Hyperparameter tuning ranges***

Machine learned model optimization was automated with support from Amazon SageMaker Autopilot [25]. Hyperparameter tuning used Bayesian optimization [26] and Amazon SageMaker Automatic Model Tuning [27]. Hyperparameter tuning ranges were developed for each modelling framework (Table S5). Some hyperparameters specific to each framework were not optimized but were instead fixed for all applicable models. For XGBoost models this included 5-fold cross validation (multiple splits of training and testing data to compare models optimized using different data which tends to make model output more robust to novel data). All LinearLearner models used 800 samples at each iteration. The multi-layered perceptron used batch normalization (centering and standardizing each feature), a rectified linear activation function (a function that is linear for positive inputs and zero for negative inputs), 10 warm-up epochs (initial training epochs that use a small learning rate to assist adaptive optimizers), and 3 rounds of 5-fold cross validation.

***Confusion matrices***

Confusion matrices were constructed from results from each candidate model pipeline evaluated using all 3 feature sets (Movement and Timing, History, and Habitat) and for the best performing model pipeline for 3 reduced combinations of available feature sets (Movement and Timing and History; Movement and Timing and Habitat; Movement and Timing only; Tables S5-S17).

***Supplementary Information References***

1. McDuie F, Casazza ML, Overton CT, Herzog MP, Hartman CA, Peterson SH, et al. GPS tracking data reveals daily spatio-temporal movement patterns of waterfowl. Mov Ecol. 2019;7:6.
2. McDuie F, Lorenz AA, Klinger RC, Overton CT, Feldheim CL, Ackerman JT, et al. Informing wetland management with waterfowl movement and sanctuary use responses to human-induced disturbance. J Environ Manage. 2021;297:113170.
3. Croston R, Hartman CA, Herzog MP, Casazza ML, Feldheim CL, Ackerman JT. Timing, frequency, and duration of incubation recesses in dabbling ducks. Ecol Evol. 2020;10:2513–29.
4. Peterson SH, Ackerman JT, Herzog MP, Hartman CA, Croston R, Feldheim CL, et al. Sitting ducklings: Timing of hatch, nest departure, and predation risk for dabbling duck broods. Ecol Evol. 2019;9:5490–500.
5. Shamoun-Baranes J, Bom R, van Loon EE, Ens BJ, Oosterbeek K, Bouten W. From Sensor Data to Animal Behaviour: An Oystercatcher Example. PLoS ONE. 2012;7:e37997.
6. Kohl JD. Identifying Postbreeding Molting Sites and Factors Influencing Molting Chronology for Gadwall (Mareca strepera) and Mallards (Anas platyrhynchos) Nesting in the Suisun Marsh of California [PhD Thesis]. University of California, Davis; 2019.
7. Roever CL, Beyer HL, Chase MJ, van Aarde RJ. The pitfalls of ignoring behaviour when quantifying habitat selection. Diversity Distrib. 2014;20:322–33.
8. R Core Team. R: A Language and Environment for Statistical Computing. R Foundation for Statistical Computing, Vienna, Austria: Available at: https://www R-project org/[Google Scholar]. 2019;
9. Croston R, Peterson SH, Hartman CA, Herzog MP, Feldheim CL, Casazza ML, et al. Nocturnal incubation recess and flushing behavior by duck hens. Ecol Evol. 2021;11:7292–301.
10. McNeil R, Drapeau P, Goss-Custard JD. The occurrence and adaptive significance of nocturnal habits in waterfowl. Biol Rev. 1992;67:381–419.
11. Gorelick N, Hancher M, Dixon M, Ilyushchenko S, Thau D, Moore R. Google Earth Engine: Planetary-scale geospatial analysis for everyone. Remote Sens Environ. 2017;202:18–27.
12. Du Y, Zhang Y, Ling F, Wang Q, Li W, Li X. Water Bodies’ Mapping from Sentinel-2 Imagery with Modified Normalized Difference Water Index at 10-m Spatial Resolution Produced by Sharpening the SWIR Band. Remote Sens-Basel. 2016;8:354.
13. Wickham H, Hester J, Francois R. readr: Read Rectangular Text Data [Internet]. 2018. Available from: https://CRAN.R-project.org/package=readr
14. Wickham H, François R, Henry L, Müller K. dplyr: A Grammar of Data Manipulation [Internet]. 2021. Available from: https://CRAN.R-project.org/package=dplyr
15. Signer J, Fieberg J, Avgar T. Animal movement tools ( amt ): R package for managing tracking data and conducting habitat selection analyses. Ecol Evol. 2019;9:880–90.
16. Grolemund G, Wickham H. Dates and Times Made Easy with lubridate. J Stat Softw. 2011;40:1–25.
17. Hijmans RJ. geosphere: Spherical Trigonometry [Internet]. 2019. Available from: https://CRAN.R-project.org/package=geosphere
18. Bivand R, Lewin-Koh N. maptools: Tools for Handling Spatial Objects [Internet]. 2019. Available from: https://CRAN.R-project.org/package=maptools
19. Bivand R, Keitt T, Rowlingson B. rgdal: Bindings for the “Geospatial” Data Abstraction Library [Internet]. 2021. Available from: https://CRAN.R-project.org/package=rgdal
20. Wickham H. tidyr: Tidy Messy Data [Internet]. 2021. Available from: https://CRAN.R-project.org/package=tidyr
21. Wickham H, Bryan J. readxl: Read Excel Files [Internet]. 2019. Available from: https://CRAN.R-project.org/package=readxl
22. Pebesma E. Simple Features for R: Standardized Support for Spatial Vector Data. The R Journal. 2018;10(1):439.
23. Ushey K, Allaire JJ, Wickham H, Ritchie G. rstudioapi: Safely Access the RStudio API [Internet]. 2020. Available from: https://CRAN.R-project.org/package=rstudioapi
24. Wickham H. stringr: Simple, Consistent Wrappers for Common String Operations [Internet]. 2019. Available from: https://CRAN.R-project.org/package=stringr
25. Das P, Ivkin N, Bansal T, Rouesnel L, Gautier P, Karnin Z, et al. Amazon SageMaker Autopilot: a white box AutoML solution at scale. In: Proceedings of the Fourth International Workshop on Data Management for End-to-End Machine Learning [Internet]. Portland OR USA: ACM; 2020 [cited 2021 Aug 28]. p. 1–7. Available from: https://dl.acm.org/doi/10.1145/3399579.3399870
26. Snoek J, Larochelle H, Adams RP. Practical bayesian optimization of machine learning algorithms. Adv Neur In. 2012;25.
27. Perrone V, Shen H, Zolic A, Shcherbatyi I, Ahmed A, Bansal T, et al. Amazon SageMaker Automatic Model Tuning: Scalable Gradient-Free Optimization. arXiv:201208489 [cs, stat] [Internet]. 2021 Jun 18 [cited 2021 Sep 17]; Available from: http://arxiv.org/abs/2012.08489

Table S1. We identified 13 common steps used during supervised classification with machine learned models. Automated data pipelines generated code to complete more than half of those steps (in bold). We elected to not host the best performing model on-line to reduce the maintenance costs related to cloud-computing.

| 1. Data Collection |
| --- |
| 1. Data Verification – QA/QC |
| 1. Data Labelling – known class assignment (similar to dependent variable identification) |
| 1. Data Augmentation – synthesizing new data from existing data |
| 1. Feature Development – characteristics or summary metrics of predictive information (similar to independent variable creation in statistics) |
| 1. **Choosing a Modelling Framework (e.g., Support Vector Machine, Extreme Gradient Boosted Classifier, Multi-Layer Perceptron)** |
| 1. **Feature Preprocessing (e.g., indicator variable or one-hot encoding, feature scaling)** |
| 1. **Model Training - optimizes the best combination and parameterization of input features given known classes** |
| 1. **Model Validation - application of the trained model to data randomly withheld from training (prevents overfitting and provides a less biased estimate of model performance)** |
| 1. **Hyperparameter Tuning - optimizes model architecture and/or parameters that govern how a model improves (i.e., “learns”) across repeated iterations** |
| 1. Model testing – using a second data set randomly withheld from training and testing for an unbiased final interpretation of model performance |
| 1. **Model Evaluation – contrast results from alternate candidate models to identify the best performing classifier** |
| 1. **Endpoint Hosting – staging a model for future classification of novel data, hosting can be local or remote (“in the cloud”) to enable near real time-classification** |

Table S2. Engineered features comprising the Movement and Timing feature set

| **Feature Description** |  |
| --- | --- |
| Sine of day of year | |
| Cosine of day of year | |
| Logarithm of mean distance between locations | |
| Logarithm of median distance between locations | |
| Logarithm of maximum distance between locations | |
| Proportion of sequential movements less than 100m | |
| Proportion of sequential movements less than 300m | |
| Proportion of sequential movements less than 500m | |
| Proportion of sequential movements less than 1km | |
| Proportion of sequential movements less than 10km | |
| Proportion of sequential movements less than 25km | |
| Proportion of sequential movements less than 50km | |
| Proportion of sequential movements less than 100km | |
| Standard deviation of movement from each location to all subsequent locations in a day | |
| Standard deviation of movement from each location to the successive location | |
| Mean observed speed through the day | |
| Median observed speed through the day | |
| Maximum observed speed through the day | |
| Number of movements greater than 5 kilometers per hour | |
| Number of movements greater than 10 kilometers per hour | |
| Number of movements greater than 25 kilometers per hour | |
| Number of movements greater than 50 kilometers per hour | |
| Average number of locations within 50 meters of each location | |
| Average number of locations within 50 meters of each location during the nighttime | |
| Logarithm of median distance between each daytime to each nighttime location | |
| Logarithm of mean distance between each daytime to each nighttime location | |
| Logarithm of median distance among all daytime locations | |
| Logarithm of median distance among all nighttime locations | |
| Logarithm of distance between first and last location in a day | |
| Logarithm of distance between first location and the location at the middle of the day | |
| Logarithm of distance between the location at the middle of the day and the last location of the day | |
| Ratio of maximum distance moved and median distance moved among all locations | |
| Proportion of overlap between the daily 25-percentile and 75-percentile 30-meter radii local convex hulls | |
| Proportion of overlap between the daily 50-percentile and 100-percentile 30-meter radii local convex hulls | |
| Number of daily hulls identified using 30-meter radii local convex hull (100-percentile hulls) | |
| Number of focal area hulls identified using 30-meter radii local convex hull (used for more than 3 hours) | |
| Logarithm of maximum distance between 30-meter radii local convex hulls | |
| Logarithm of mean distance between 30-meter radii local convex hulls | |
| Logarithm of maximum distance between 30-meter radii local convex hulls used for more than 3 hours | |
| Logarithm of mean distance between 30-meter radii local convex hulls used for more than 3 hours | |

Table S3. Engineered features comprising the History feature set

| **Feature Description** |  |
| --- | --- |
| Mean distance between locations on target date and locations on previous day | |
| Mean distance between locations on target date and locations 2-3 days prior | |
| Mean distance between locations on target date and locations 5-7 days prior | |
| Mean distance between locations on target date and locations 8-10 days prior | |
| Mean distance between locations on target date and locations 12-15 days prior | |
| Proportion of distances between locations on target date locations on previous day that are within 100m | |
| Proportion of distances between locations on target date locations 2-3 days prior that are within 100m | |
| Proportion of distances between locations on target date locations 5-7 days prior that are within 100m | |
| Proportion of distances between locations on target date locations 8-10 days prior that are within 100m | |
| Proportion of distances between locations on target date locations 12-15 days prior that are within 100m | |
| Proportion of distances between locations on target date locations on previous day that are within 500m | |
| Proportion of distances between locations on target date locations 2-3 days prior that are within 500m | |
| Proportion of distances between locations on target date locations 5-7 days prior that are within 500m | |
| Proportion of distances between locations on target date locations 8-10 days prior that are within 500m | |
| Proportion of distances between locations on target date locations 12-15 days prior that are within 500m | |
| Proportion of distances between locations on target date locations on previous day that are within 25k | |
| Proportion of distances between locations on target date locations 2-3 days prior that are within 25k | |
| Proportion of distances between locations on target date locations 5-7 days prior that are within 25k | |
| Proportion of distances between locations on target date locations 8-10 days prior that are within 25k | |
| Proportion of distances between locations on target date locations 12-15 days prior that are within 25k | |

Table S4. Engineered features comprising the Habitat feature set

| **Feature Description** |
| --- |
| Mean modified normalized difference water index (MNDWI) at observed locations |
| Median MNDWI at observed locations |
| Number of locations with MNDWI < 0 |
| Number of locations with MNDWI >= 0 |
| Number of locations with MNDWI < -0.3 |
| Number of locations with MNDWI between 0 and -0.3 |
| Lower quartile of MNDWI at observed locations |
| Upper quartile of MNDWI at observed locations |

Table S5. Tuning range values for hyperparameters by model framework.

| XGBoost | Hyperparameter | Minimum | |  | Maximum | | Scale |
| --- | --- | --- | --- | --- | --- | --- | --- |
|  | Number of rounds of training | 2 | |  | 1024 | | Logarithmic |
|  | Maximum depth of tree | 2 | |  | 8 | | Logarithmic |
|  | Step size shrinkage | 0.001 | |  | 1 | | Logarithmic |
|  | Minimum loss reduction to partition tree | 0.000001 | |  | 64 | | Logarithmic |
|  | Minimum hessian needed in node | 0.000001 | |  | 32 | | Logarithmic |
|  | Ratio of data to subsample per instance | 0.5 | |  | 1 | | Linear |
|  | Subsample ratio of columns for tree | 0.3 | |  | 1 | | Linear |
|  | L2 regularization term | 0.000001 | |  | 2 | | Logarithmic |
|  | L1 regularization term | 0.000001 | |  | 2 | | Logarithmic |
|  |  |  | |  |  | |  |
| Linear Learner | L2 regularization term | 0.0000001 | |  | 1 | | Logarithmic |
|  | L1 regularization term | 0.0000001 | |  | 1 | | Logarithmic |
|  | learning rate | 0.00001 | |  | 1 | | Logarithmic |
|  |  |  | |  |  | |  |
| Multilayer Perceptron | Minimum batch size | 128 | |  | 512 | | Linear |
|  | Learning rate | 0.000001 | |  | 0.01 | | Logarithmic |
|  | L2 regularization term | 1.00E-12 | |  | 0.01 | | Logarithmic |
|  | Dropout probability | 0.25 | |  | 0.5 | | Linear |
|  | Embedding size factor | 0.65 | |  | 0.95 | | Linear |
|  | Network type | Feed-forward | |  | Wide-deep | | Categorical |
|  | Layer structure | 256 | 50 | | | 25 | Categorical |
|  |  | 100 | 50 | | |  |  |
|  |  | 200 | 100 | | |  |  |
|  |  | 256 | 128 | | |  |  |
|  |  | 300 | 150 | | |  |  |
|  |  | 200 | 100 | | | 50 |  |

Table S6. Confusion matrix from optimized model #1 using the XGBoost framework (best performing model) and using all 3 feature sets.

|  |  |  | Predicted Class | |  |  |  |  |
| --- | --- | --- | --- | --- | --- | --- | --- | --- |
| Actual Class | Brooding | Dead | Local | Migration | Molt-Like | Molting | Nesting | Regional Relocation |
| Brooding | 8 | 0 | 2 | 0 | 11 | 0 | 0 | 0 |
| Dead | 0 | 189 | 0 | 0 | 0 | 0 | 0 | 0 |
| Local | 0 | 0 | 839 | 0 | 20 | 0 | 0 | 3 |
| Migration | 0 | 0 | 0 | 19 | 0 | 0 | 0 | 1 |
| Molt-Like | 0 | 0 | 27 | 0 | 561 | 3 | 2 | 0 |
| Molting | 0 | 0 | 0 | 0 | 14 | 73 | 0 | 0 |
| Nesting | 0 | 0 | 2 | 0 | 5 | 0 | 51 | 0 |
| Regional Relocation | 0 | 0 | 0 | 0 | 0 | 0 | 0 | 37 |

Table S7. Confusion matrix from optimized model #2 using the Linear Learner (Stochastic Gradient Decent) framework and using all 3 feature sets.

|  |  |  | Predicted Class | |  |  |  |  |
| --- | --- | --- | --- | --- | --- | --- | --- | --- |
| Actual Class | Brooding | Dead | Local | Migration | Molt-Like | Molting | Nesting | Regional Relocation |
| Brooding | 3 | 0 | 2 | 0 | 15 | 0 | 1 | 0 |
| Dead | 0 | 187 | 0 | 0 | 1 | 0 | 1 | 0 |
| Local | 0 | 0 | 799 | 0 | 55 | 0 | 4 | 4 |
| Migration | 0 | 0 | 0 | 18 | 0 | 0 | 0 | 2 |
| Molt-Like | 6 | 0 | 52 | 1 | 499 | 30 | 5 | 0 |
| Molting | 0 | 0 | 2 | 0 | 51 | 34 | 0 | 0 |
| Nesting | 0 | 0 | 8 | 0 | 5 | 0 | 45 | 0 |
| Regional Relocation | 0 | 0 | 8 | 1 | 0 | 0 | 0 | 28 |

Table S8. Confusion matrix from optimized model #3 using the Linear Learner (Stochastic Gradient Decent) framework and using all 3 feature sets.

|  |  |  | Predicted Class | |  |  |  |  |
| --- | --- | --- | --- | --- | --- | --- | --- | --- |
| Actual Class | Brooding | Dead | Local | Migration | Molt-Like | Molting | Nesting | Regional Relocation |
| Brooding | 4 | 0 | 2 | 0 | 15 | 0 | 0 | 0 |
| Dead | 0 | 188 | 0 | 0 | 0 | 0 | 1 | 0 |
| Local | 0 | 0 | 792 | 0 | 59 | 0 | 6 | 5 |
| Migration | 0 | 0 | 0 | 15 | 0 | 0 | 0 | 5 |
| Molt-Like | 2 | 0 | 54 | 0 | 504 | 28 | 5 | 0 |
| Molting | 0 | 0 | 2 | 0 | 47 | 38 | 0 | 0 |
| Nesting | 0 | 0 | 7 | 0 | 5 | 0 | 46 | 0 |
| Regional Relocation | 0 | 0 | 4 | 1 | 0 | 0 | 0 | 32 |

Table S9. Confusion matrix from optimized model #4 using the XGBoost framework and using all 3 feature sets.

|  |  |  | Predicted Class | |  |  |  |  |
| --- | --- | --- | --- | --- | --- | --- | --- | --- |
| Actual Class | Brooding | Dead | Local | Migration | Molt-Like | Molting | Nesting | Regional Relocation |
| Brooding | 8 | 0 | 2 | 0 | 11 | 0 | 0 | 0 |
| Dead | 0 | 189 | 0 | 0 | 0 | 0 | 0 | 0 |
| Local | 0 | 0 | 839 | 0 | 20 | 0 | 0 | 3 |
| Migration | 0 | 0 | 0 | 20 | 0 | 0 | 0 | 0 |
| Molt-Like | 0 | 0 | 30 | 0 | 557 | 4 | 2 | 0 |
| Molting | 0 | 0 | 0 | 0 | 18 | 69 | 0 | 0 |
| Nesting | 0 | 0 | 2 | 0 | 5 | 0 | 51 | 0 |
| Regional Relocation | 0 | 0 | 1 | 0 | 0 | 0 | 0 | 36 |

Table S10. Confusion matrix from optimized model #5 using the Linear Learner (Stochastic Gradient Decent) framework and using all 3 feature sets.

|  |  |  | Predicted Class | |  |  |  |  |
| --- | --- | --- | --- | --- | --- | --- | --- | --- |
| Actual Class | Brooding | Dead | Local | Migration | Molt-Like | Molting | Nesting | Regional Relocation |
| Brooding | 2 | 0 | 1 | 0 | 17 | 0 | 1 | 0 |
| Dead | 0 | 187 | 0 | 0 | 0 | 0 | 2 | 0 |
| Local | 0 | 0 | 801 | 0 | 52 | 3 | 1 | 5 |
| Migration | 0 | 0 | 0 | 14 | 1 | 0 | 0 | 5 |
| Molt-Like | 4 | 1 | 71 | 0 | 478 | 33 | 5 | 1 |
| Molting | 0 | 0 | 1 | 0 | 39 | 47 | 0 | 0 |
| Nesting | 0 | 0 | 11 | 0 | 5 | 0 | 42 | 0 |
| Regional Relocation | 0 | 0 | 5 | 0 | 0 | 1 | 1 | 30 |

Table S11. Confusion matrix from optimized model #6 using the Linear Learner (Stochastic Gradient Decent) framework and using all 3 feature sets.

|  |  |  | Predicted Class | |  |  |  |  |
| --- | --- | --- | --- | --- | --- | --- | --- | --- |
| Actual Class | Brooding | Dead | Local | Migration | Molt-Like | Molting | Nesting | Regional Relocation |
| Brooding | 1 | 0 | 2 | 0 | 18 | 0 | 0 | 0 |
| Dead | 0 | 185 | 0 | 0 | 1 | 2 | 1 | 0 |
| Local | 0 | 0 | 764 | 0 | 83 | 5 | 5 | 5 |
| Migration | 0 | 0 | 2 | 16 | 0 | 0 | 0 | 2 |
| Molt-Like | 4 | 4 | 86 | 0 | 472 | 22 | 5 | 0 |
| Molting | 0 | 1 | 4 | 0 | 49 | 33 | 0 | 0 |
| Nesting | 0 | 2 | 13 | 0 | 9 | 0 | 34 | 0 |
| Regional Relocation | 0 | 0 | 11 | 4 | 0 | 0 | 0 | 22 |

Table S12. Confusion matrix from optimized model #7 using the Linear Learner (Stochastic Gradient Decent) framework and using all 3 feature sets.

|  |  |  | Predicted Class | |  |  |  |  |
| --- | --- | --- | --- | --- | --- | --- | --- | --- |
| Actual Class | Brooding | Dead | Local | Migration | Molt-Like | Molting | Nesting | Regional Relocation |
| Brooding | 2 | 0 | 2 | 0 | 17 | 0 | 0 | 0 |
| Dead | 0 | 187 | 0 | 0 | 1 | 0 | 1 | 0 |
| Local | 1 | 0 | 791 | 0 | 56 | 2 | 6 | 6 |
| Migration | 0 | 0 | 1 | 19 | 0 | 0 | 0 | 0 |
| Molt-Like | 4 | 2 | 66 | 0 | 495 | 19 | 7 | 0 |
| Molting | 0 | 0 | 1 | 0 | 48 | 37 | 1 | 0 |
| Nesting | 0 | 0 | 13 | 0 | 7 | 0 | 38 | 0 |
| Regional Relocation | 0 | 0 | 7 | 2 | 0 | 0 | 1 | 27 |

Table S13. Confusion matrix from optimized model #8 using the XGBoost framework and using all 3 feature sets.

|  |  |  | Predicted Class | |  |  |  |  |
| --- | --- | --- | --- | --- | --- | --- | --- | --- |
| Actual Class | Brooding | Dead | Local | Migration | Molt-Like | Molting | Nesting | Regional Relocation |
| Brooding | 8 | 0 | 2 | 0 | 11 | 0 | 0 | 0 |
| Dead | 0 | 189 | 0 | 0 | 0 | 0 | 0 | 0 |
| Local | 0 | 0 | 839 | 0 | 20 | 0 | 0 | 3 |
| Migration | 0 | 0 | 0 | 20 | 0 | 0 | 0 | 0 |
| Molt-Like | 0 | 0 | 30 | 0 | 557 | 4 | 2 | 0 |
| Molting | 0 | 0 | 0 | 0 | 18 | 69 | 0 | 0 |
| Nesting | 0 | 0 | 2 | 0 | 5 | 0 | 51 | 0 |
| Regional Relocation | 0 | 0 | 1 | 0 | 0 | 0 | 0 | 36 |

Table S14. Confusion matrix from optimized model #9 using the XGBoost framework and using all 3 feature sets.

|  |  |  | Predicted Class | |  |  |  |  |
| --- | --- | --- | --- | --- | --- | --- | --- | --- |
| Actual Class | Brooding | Dead | Local | Migration | Molt-Like | Molting | Nesting | Regional Relocation |
| Brooding | 8 | 0 | 2 | 0 | 11 | 0 | 0 | 0 |
| Dead | 0 | 189 | 0 | 0 | 0 | 0 | 0 | 0 |
| Local | 0 | 0 | 839 | 0 | 20 | 0 | 0 | 3 |
| Migration | 0 | 0 | 0 | 20 | 0 | 0 | 0 | 0 |
| Molt-Like | 0 | 0 | 30 | 0 | 557 | 4 | 2 | 0 |
| Molting | 0 | 0 | 0 | 0 | 17 | 70 | 0 | 0 |
| Nesting | 0 | 0 | 2 | 0 | 5 | 0 | 51 | 0 |
| Regional Relocation | 0 | 0 | 0 | 0 | 0 | 0 | 0 | 37 |

Table S15. Confusion matrix from optimized model #10 using the Multilayer Perceptron framework and using all 3 feature sets.

|  |  |  | Predicted Class | |  |  |  |  |
| --- | --- | --- | --- | --- | --- | --- | --- | --- |
| Actual Class | Brooding | Dead | Local | Migration | Molt-Like | Molting | Nesting | Regional Relocation |
| Brooding | 6 | 0 | 1 | 0 | 14 | 0 | 0 | 0 |
| Dead | 0 | 189 | 0 | 0 | 0 | 0 | 0 | 0 |
| Local | 0 | 0 | 815 | 0 | 36 | 0 | 6 | 5 |
| Migration | 0 | 0 | 0 | 19 | 0 | 0 | 0 | 1 |
| Molt-Like | 5 | 1 | 26 | 0 | 550 | 8 | 3 | 0 |
| Molting | 1 | 0 | 0 | 0 | 26 | 60 | 0 | 0 |
| Nesting | 0 | 0 | 4 | 0 | 4 | 0 | 50 | 0 |
| Regional Relocation | 0 | 0 | 1 | 0 | 0 | 0 | 0 | 36 |

Table S16. Confusion matrix from optimized model #1 using the XGBoost framework (best performing model) and using Movement and Timing and History feature sets only.

|  |  |  | Predicted Class | |  |  |  |  |
| --- | --- | --- | --- | --- | --- | --- | --- | --- |
| Actual Class | Brooding | Dead | Local | Migration | Molt-Like | Molting | Nesting | Regional Relocation |
| Brooding | 7 | 0 | 2 | 0 | 12 | 0 | 0 | 0 |
| Dead | 0 | 189 | 0 | 0 | 0 | 0 | 0 | 0 |
| Local | 0 | 0 | 835 | 0 | 24 | 0 | 0 | 3 |
| Migration | 0 | 0 | 0 | 19 | 0 | 0 | 0 | 1 |
| Molt-Like | 0 | 1 | 28 | 0 | 553 | 10 | 1 | 0 |
| Molting | 0 | 0 | 0 | 0 | 27 | 60 | 0 | 0 |
| Nesting | 0 | 0 | 2 | 0 | 8 | 0 | 48 | 0 |
| Regional Relocation | 0 | 0 | 1 | 0 | 0 | 0 | 0 | 36 |

Table S17. Confusion matrix from optimized model #1 using the XGBoost framework (best performing model) and using Movement and Timing and Habitat feature sets only.

|  |  |  | Predicted Class | |  |  |  |  |
| --- | --- | --- | --- | --- | --- | --- | --- | --- |
| Actual Class | Brooding | Dead | Local | Migration | Molt-Like | Molting | Nesting | Regional Relocation |
| Brooding | 3 | 0 | 2 | 0 | 16 | 0 | 0 | 0 |
| Dead | 0 | 188 | 0 | 0 | 1 | 0 | 0 | 0 |
| Local | 0 | 0 | 825 | 0 | 33 | 0 | 1 | 3 |
| Migration | 0 | 0 | 0 | 18 | 0 | 0 | 0 | 2 |
| Molt-Like | 1 | 2 | 36 | 0 | 550 | 3 | 1 | 0 |
| Molting | 0 | 0 | 0 | 0 | 24 | 63 | 0 | 0 |
| Nesting | 0 | 0 | 3 | 0 | 6 | 0 | 49 | 0 |
| Regional Relocation | 0 | 0 | 1 | 0 | 0 | 0 | 0 | 36 |

Table S18. Confusion matrix from optimized model #1 using the XGBoost framework (best performing model) and using the Movement and Timing feature set only.

|  |  |  | Predicted Class | |  |  |  |  |
| --- | --- | --- | --- | --- | --- | --- | --- | --- |
| Actual Class | Brooding | Dead | Local | Migration | Molt-Like | Molting | Nesting | Regional Relocation |
| Brooding | 0 | 0 | 2 | 0 | 18 | 0 | 1 | 0 |
| Dead | 0 | 187 | 0 | 0 | 2 | 0 | 0 | 0 |
| Local | 0 | 0 | 820 | 0 | 39 | 0 | 0 | 3 |
| Migration | 0 | 0 | 0 | 18 | 0 | 0 | 0 | 2 |
| Molt-Like | 1 | 2 | 41 | 0 | 528 | 19 | 2 | 0 |
| Molting | 0 | 0 | 0 | 0 | 44 | 43 | 0 | 0 |
| Nesting | 0 | 2 | 7 | 0 | 9 | 0 | 40 | 0 |
| Regional Relocation | 0 | 0 | 2 | 1 | 0 | 0 | 0 | 34 |

Additional File 2:

**Feature Engineering Script for Daily Activity Classification of Waterfowl Life History States**

Language: R

See “Supplemental File 2 R Code for feature engineering.R”

Input requirements: 3 Input files (comma delimited text)

Pre-deployment locations were used in a few cases to approximate mortality. This code identifies those locations and processes them separate from data on deployed individuals. Operational use of this code could be rewritten to omit those steps which would not be relevant when processing only deployed GPS locations. Following completion of these scripts, output files were loaded into AWS S3 locations for preprocessing (see Supplementary File 3).

File 1: Named “AllLocsReducedColumns.csv” contains all locations collected regardless of frequency and completeness, used to calculate spatial position of target date locations to prior day locations. Requires 6 fields:

individual_local_identifier <character> : Unique identifier for GPS marked individual

tag_local_identifier <character> : Identifier for Transmitter

individual_taxon_canonical_name <character> : Taxon name

location_lat <numeric> : WGS84 latitude in decimal degrees

location_long <numeric> : WGS84 longitude in decimal degrees

timestamp <POSIXct> : datetime in UTC

File 2: Named “testMB2animals.csv” contains deployment information linking transmitters to specific individuals. Requires 4 fields:

local_identifier <character> : Unique identifier for GPS marked individual

taxon_canonical_name <character> : Taxon name

timestamp_start <POSIXct> : Start of active deployment

timestamp_end <POSIXct> : End of active deployment

File 3: Named “FinalDate_and_Status_List_Full_Data_withfinalclass_MNDWI.csv” contains data annotated with Life History State and includes assessments of monthly habitat condition at locations generated via Google Earth Engine and using Sentinel 2, Landsat 7 and Landsat 8 satellite imagery. Requires 12 Fields.

‘Xdeployment ID’ <character> : Unique identifier for GPS marked individual

Concat <character> : Concatenation of ‘Xdeployment ID’ and Timestamp. Unique identifier for location

Timestamp <POSIXct> : datetime in UTC

Location Order <numeric> : sequential ID of sorted relocations by individual

Status <character> : Annotated/labelled life history state

birddate <character> : Concatenation of ‘Xdeployment ID’ and Date represented in Timestamp. Uniquely identifies each set of 24 hourly relocations for classification

ydate <numeric> : Day of year

year <numeric> : Year

MNDWI <numeric> : Mean Modified Normalized Difference Water Index at the location coordinates calculated from all Sentinel 2, Landsat 7 or Sentinel 8 satellite images collected in the same month as the location

wet_or_dry <character> : Categorical indicator for habitat condition. MNDWI greater than or equal to 0 are “Wet” and less than 0 are “Dry”

Location-Lat <numeric> : WGS84 latitude in decimal degrees

Location-Long <numeric> : WGS84 longitude in decimal degrees

Additional File 3:

**Data preprocessing, testing data splitting and preparation for AWS SageMaker Studio**

See “Supplemental File 3 duck-lifestage-classification-datasplit.ipynb”

Language: Python

Input requirements: 1 Input files (comma delimited text) filename = 'augmentedhouryset_analysisreadywithhabitatandpastlocations_wideformat_withID.csv' that is output from R code and loaded into an S3 bucket with the tag “rawdata”

After the file containing features to be processed are loaded into AWS, this code will process it into multiple formats for different possible SageMaker workflows. This code also subsets those features retained for individual feature set assessments used in this manuscript. These may not be necessary for operational use. Following completion of this code we ran an AWS SageMaker Studio Experiment to complete the classification using the Automated Modelling Pipeline.

Additional File 4:

**Automated Modeling Pipeline Code**

See “Supplemental File 4 automl code DuckLifestageClass-all.ipynb”

Language: Python

Input requirements: 1 Input file (comma delimited text) output from preprocessing Python Notebook run in AWS SageMaker.

This is the AWS automatically generated code that defined candidate models to classify daily activity reflecting life history states using hourly GPS and habitat data. This code was entirely created by the automated modelling pipeline. Within the code AWS SageMaker chose 3 modelling frameworks for multiclassification problems, developed alternative feature preprocessing steps for each of 10 candidate models, trained then validated each model and tuned hyperparameters to optimize performance. The best model among all candidates was identified and it provides capacity to host endpoints for classification of novel data.
